# Telocytes constitute a widespread interstitial meshwork in the lamina propria and underlying striated muscle of human tongue

**DOI:** 10.1038/s41598-019-42415-3

**Published:** 2019-04-10

**Authors:** Irene Rosa, Cecilia Taverna, Luca Novelli, Mirca Marini, Lidia Ibba-Manneschi, Mirko Manetti

**Affiliations:** 1 Department of Experimental and Clinical Medicine, Section of Anatomy and Histology, University of Florence, Florence, Italy; 2 Institute of Histopathology and Molecular Diagnosis, Careggi University Hospital, Florence, Italy

## Abstract

Telocytes have recently emerged as unique interstitial cells defined by their extremely long, thin and moniliform prolongations termed telopodes. Despite growing evidence that these cells consistently reside in the stromal compartment of various organs from human beings, studies dealing with telocytes in structures of the oral cavity are scarce. Hence, the present morphologic study was undertaken to explore for the first time the presence and specific localization of telocytes within tissues of the normal human tongue, a complex muscular organ whose main functions include taste, speech, and food manipulation in the oral cavity. Telocytes were initially identified by CD34 immunostaining and confirmed by CD34/PDGFRα double immunofluorescence and transmission electron microscopy. CD34+/PDGFRα+ telocytes were organized in interstitial meshworks either in the tongue lamina propria or in the underlying striated muscle. Lingual telocytes were immunonegative for CD31, c-kit and α-SMA. Telopodes were finely distributed throughout the stromal space and concentrated beneath the lingual epithelium and around CD31+ vessels, skeletal muscle bundles/fibers, and intramuscular nerves and ganglia. They also enveloped salivary gland units outside the α-SMA+ myoepithelial cells and delimited lymphoid aggregates. These findings establish telocytes as a previously overlooked interstitial cell population worth investigating further in the setting of human tongue pathophysiology.

## Introduction

During the last decade, there has been substantial progress in our understanding of the morphofunctional characteristics of a variety of organs from different systems thanks to the discovery of a previously neglected cell population residing in the stromal space, namely telocytes (TCs)^1–6^. These cells have been primarily recognized based on their unique ultrastructural morphology as interstitial/stromal cells featuring a small nucleated cell body giving rise to exceptionally long moniliform/varicose cytoplasmic processes (termed ‘telopodes’) with a ‘beads-on-a-string’-like appearance conferred by the alternation of very slender segments and cistern-like dilated portions (termed ‘podomers’ and ‘podoms’, respectively)^1–4,7–10^. Afterwards, many efforts have been made to further differentiate TCs from other stromal cell types, mainly fibroblasts, through an in-depth deciphering of their immunophenotypic features and gene expression, proteomic and microRNA profiles^1,2,11–15^. Though at present TC-specific antigens are still lacking, TCs from virtually every organs (at least in humans) appear to consistently express the cell surface glycoprotein CD34 and, therefore, they are commonly referred to also as TCs/CD34+ stromal cells^1,4,9,10,16–18^. Furthermore, mounting evidence indicates that colocalization of CD34 and platelet-derived growth factor receptor α (PDGFRα, also known as CD140a) is a hallmark of TCs, making them straightforwardly discernible by means of CD34/PDGFRα double immunofluorescence staining^1,2,9,10,19–22^. Telopodes are diffusely distributed and interconnected in the interstitium where they organize three-dimensionally in a labyrinthine meshwork communicating and exchanging signals with a variety of adjacent cell types through both cell-to-cell connections and release of extracellular vesicles^1,2,23–29^. As exceptional interstitial connecting devices, TCs are supposed to be committed to intercellular signaling likely making a significant contribution to tissue morphogenetic and developmental processes, homeostatic balance, immunosurveillance as well as reparative/regenerative mechanisms by supporting local stem cell niche maintenance and differentiation or performing themselves as progenitors^1–4,30–35^. Besides tissue physiologic conditions, the notion that TCs may undergo interesting variations in a number of pathologic states suggests either their possible pathogenetic implications or their potentiality as target cells for regenerative medicine purposes^36–42^.

Thus far, TCs have been broadly described in multiple microanatomic locations, including the connective compartment of various parenchymatous organs, the lamina propria and submucosa of several hollow organs, and the interstitium of all three types of muscle^1,2,6,16^. As far as the digestive system is concerned, these cells have been uncovered in different portions of the gastrointestinal tract, but, to the best of our knowledge, reports dealing with TCs in structures of the oral cavity are scarce^6,7,16,19,21,43^. Therefore, we undertook this morphologic study to provide a first proof of the existence and microanatomic localization of TCs in tissues of the normal human tongue, a peculiar striated muscular organ covered in oral mucosa with functions as various as taste, speech, food manipulation and swallowing initiation.

## Results

**Figure 1:**
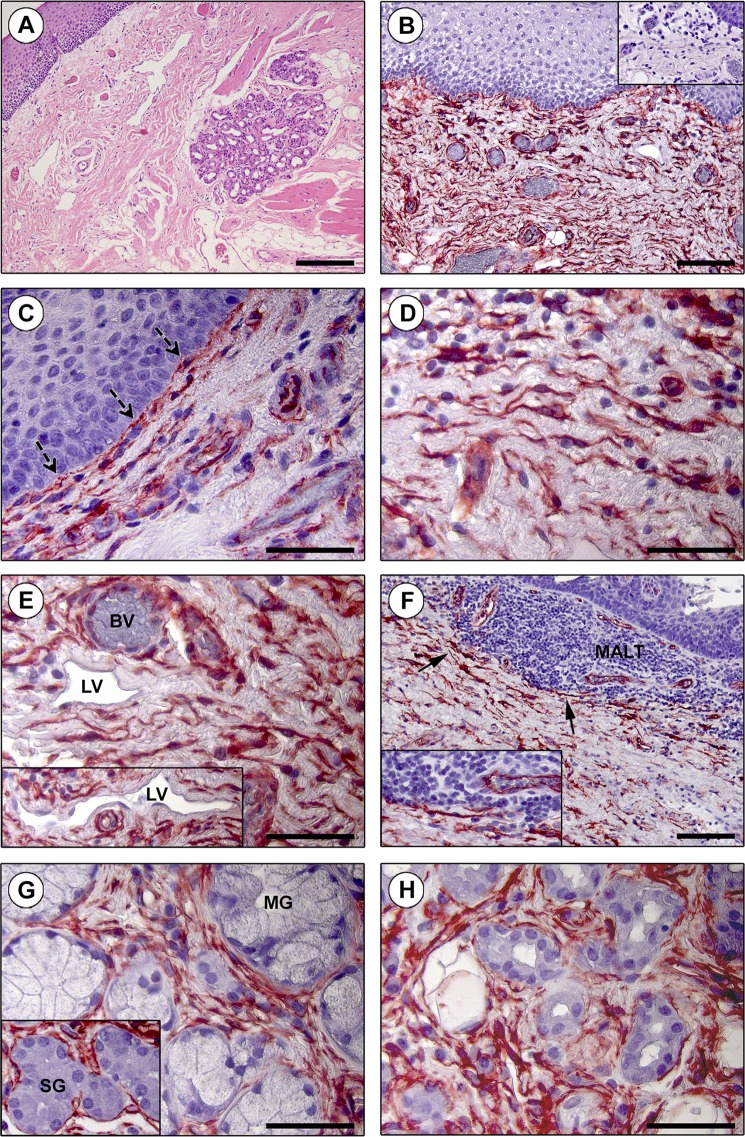
Immunohistochemical localization of telocytes (TCs)/CD34+ stromal cells in the human tongue lamina propria. (**A**) Hematoxylin and eosin staining testifying the normal appearance of the lamina propria connective tissue. (**B**–**H**) CD34 immunohistochemistry with hematoxylin counterstain. (**B**) An extensive CD34+ cell meshwork is finely distributed throughout the tongue lamina propria. Inset: negative control. (**C–E**) CD34+ interstitial cells exhibit the typical TC morphology (*i.e*. spindle-shaped cells with a small nucleated body and very long and thin moniliform/varicose telopodes). (**C**) Note the almost continuous TC layer along the basement membrane beneath the oral mucosa epithelium (dashed arrows). (**D**) TCs are in close relationship with histiocytes/mononuclear cells. (**E**) CD34+ TCs are particularly concentrated around blood vessels (BV) and lymphatic vessels (LV) (inset). CD34 immunoreactivity is detected also in blood vascular endothelium, but not in lymphatic endothelium. (**F**) TCs are arranged to delimit externally the mucosa-associated lymphoid tissue (MALT) aggregates (arrows; higher magnification in the inset). (**G**) CD34+ TCs closely surround the secretory units of mucous glands (MG) and serous glands (SG) (inset). (**H**) TCs are also distributed around salivary gland excretory ducts. Scale bar: 200 µm (**A**), 100 µm (**B,F**), 50 µm (**C–E,G,H**).

**Figure 2:**
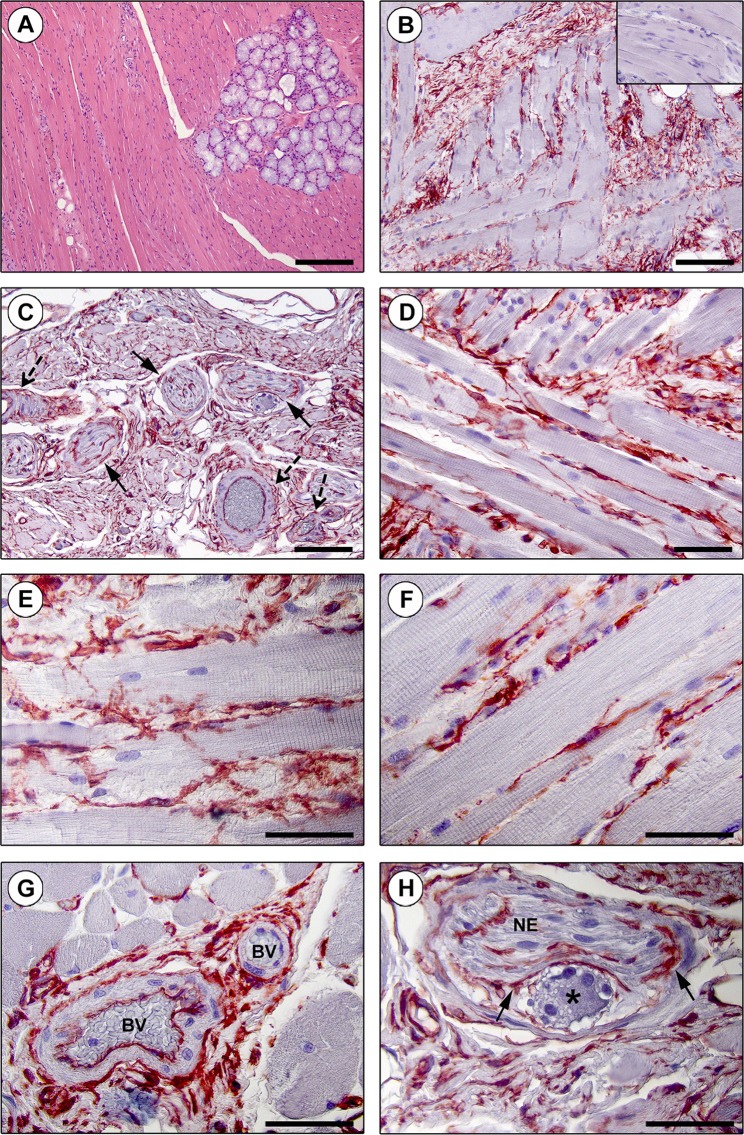
Immunohistochemical localization of telocytes (TCs)/CD34+ stromal cells in the interstitium of the human tongue striated muscle. (**A**) Hematoxylin and eosin staining demonstrating the normal appearance of the tongue muscle. (**B–H**) CD34 immunohistochemistry with hematoxylin counterstain. (**B,C**) A diffuse CD34+ reticular network is evident in the perimysium encasing skeletal muscle bundles (**B**) and around intramuscular vessels (dashed arrows) and nerves (arrows) (**C**). Inset: negative control. (**D–F**) The endomysium is populated by a dense meshwork of CD34+ TCs projecting long and moniliform telopodes in close relationship with skeletal muscle fibers. (**G**) CD34+ TCs intimately encircle intramuscular arterioles (BV, blood vessels). (**H**) TCs form an outer sheath (arrows) for intramuscular nerves (NE) and ganglia (asterisk). Scale bar: 200 µm (**A**), 100 µm (**B,C**), 50 µm (**D–H**).

In keeping with a large body of research on TCs published over the last decade^9,10,16–18,44^, we started by studying TCs within tissues of the human tongue by means of immunoperoxidase-based immunohistochemical detection of the CD34 cell surface antigen (Figs 1 and 2). To this purpose, we selected archival paraffin-embedded human tongue sections that, according to routine histologic analysis (*i.e*. hematoxylin and eosin staining), exhibited a normal microscopic structure consisting of a mass of interlacing skeletal muscle bundles firmly bound to the overlying oral mucosa by a dense connective tissue (also known as lamina propria), with numerous mucous and serous accessory salivary glands scattered throughout the tongue lamina propria and muscle (Figs 1A and 2A).

The presence and specific tissue localization of TCs/CD34+ stromal cells was investigated in detail in the whole tongue stromal compartment, namely either in the lamina propria (Fig. 1B–H) or in the interstitium of the underlying striated muscle (Fig. 2B–H).

As displayed in Fig. 1B, a low magnification overview of tissue slides highlighted the existence of an extensive and intricate CD34+ cell meshwork finely distributed in the tongue lamina propria. When observed at higher magnification, these CD34+ stromal cells demonstrated the characteristic TC morphology, that is an elongated/spindle shape with a small nucleated cell body and very long and thin moniliform/varicose prolongations (*i.e*. telopodes) (Fig. 1C–E). Immediately underneath the lingual epithelium, TCs were arranged to form a virtually continuous cell layer along the basement membrane (Fig. 1C). Moreover, in the underlying connective tissue the cytoplasmic processes of TCs were in close relationship with histiocytes/mononuclear cells and concentrated around blood and lymphatic vessels (Fig. 1D,E). CD34 immunoreactivity could also be detected in the endothelium of blood vessels, but not in lymphatic endothelial cells (Fig. 1E). As shown in Fig. 1F, TCs did not penetrate with their telopodes into mucosa-associated lymphoid tissue, though they finely delimited outwardly such subepithelial lymphoid aggregates. The interstitial TC meshwork also closely enveloped both mucous and serous secretory units and excretory ducts of lingual minor salivary glands (Fig. 1G,H).

Findings of CD34 immunohistochemistry in the tongue muscle are shown in Fig. 2B–H. The observation of immunostained slides at low magnification allowed the identification of complex CD34+ reticular networks in the perimysial connective tissue encasing the interlacing muscle bundles (Fig. 2B), as well as around intramuscular vessels and nerves (Fig. 2C). At higher magnification, a dense endomysial meshwork of CD34+ TCs projecting typical moniliform and interconnecting telopodes in the narrow interstitium between skeletal muscle fibers was further revealed (Fig. 2D–F). TCs also appeared to constitute an adventitial cell layer encircling intramuscular arterioles as well as an outer sheath for nerves and ganglia (Fig. 2G,H).

**Figure 3:**
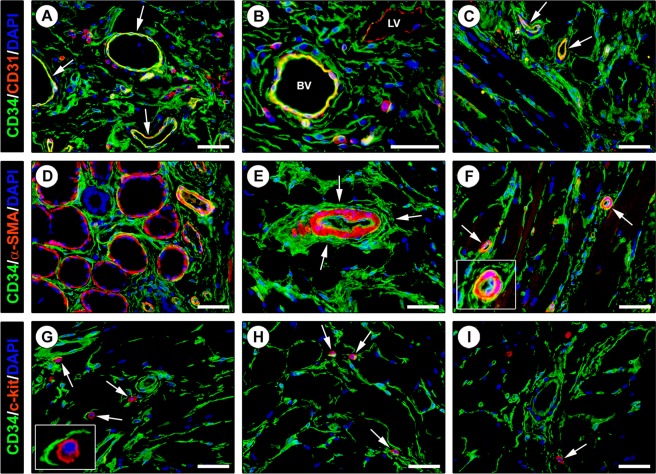
Double immunofluorescence staining of human tongue tissue sections. (**A–C**) CD34 (green) and CD31 (red) immunostaining with 4′,6-diamidino-2-phenylindole (DAPI; blue) counterstain for nuclei. Telocytes (TCs)/CD34+ stromal cells in the tongue lamina propria (**A,B**) and skeletal muscle (**C**) lack CD31 immunoreactivity. The endothelial cells of blood vessels (arrows) are CD34+/CD31+ (**A,C**). At variance with double stained blood vessels (BV), the endothelium of lymphatic vessels (LV) is CD34−/CD31+ (**B**). Some leukocytes are also CD31+ (**A,B**). (**D–F**) CD34 (green) and α-smooth muscle actin (α-SMA; red) immunofluorescence labeling with DAPI counterstain. CD34+ TCs do not coexpress α-SMA. (**D**) CD34+ TCs envelop secretory salivary gland units outside of α-SMA + myoepithelial cells. (**E,F**) CD34+ TCs externally encircle the vascular smooth muscle cell layer of arterioles (arrows; higher magnification in the inset). (**G–I**) CD34 (green) and c-kit/CD117 (red) immunofluorescence with DAPI counterstain. The CD34+ TC meshwork is immunophenotypically negative for the c-kit/CD117 marker. c-kit/CD117 is detectable only in oval/round-shaped mast cells (arrows) often in close relationship with CD34+ TC processes (higher magnification in the inset). Scale bar: 50 µm (**A–I**).

**Figure 4:**
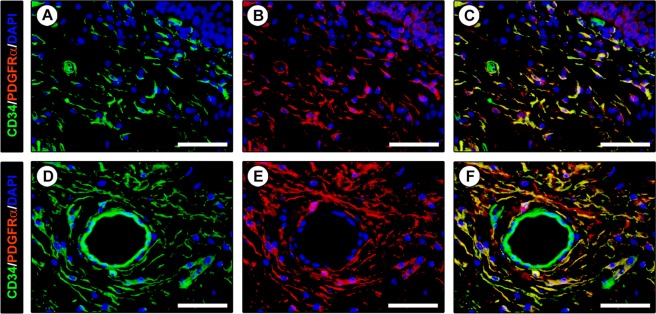
Double immunofluorescence staining of human tongue tissue sections. (**A–F**) CD34 (green) and platelet-derived growth factor receptor α (PDGFRα; red) immunofluorescence with 4′,6-diamidino-2-phenylindole (DAPI; blue) counterstain for nuclei. Single green and red images are shown in (**A,D**,**B,E**), respectively, while overlay images are shown in (**C,F**). All telocytes (TCs)/CD34+ stromal cells within the tongue stromal compartment are PDGFRα+. Colocalization of CD34 and PDGFRα on the TC surface gives rise to yellow staining. Scale bar: 50 µm (**A–F**).

In order to strengthen the afore-described findings and to gain further insights into the immunophenotypic features of lingual TCs, we next carried out a series of double immunofluorescence reactions to simultaneously detect the CD34 antigen and other markers of interest as CD31, α-smooth muscle actin (α-SMA), c-kit/CD117 and PDGFRα. According to relevant literature^6,9,10,16,17^, we first considered that CD34 cannot be assumed as a TC specific marker since it is expressed by at least another type of connective tissue-resident cells, namely CD31+ endothelial cells of blood vessels. On examination of CD34 immunolabeled tissue slides one must therefore pay attention to not misidentify as TCs even the adjacent blood vessels, particularly when the latter are sectioned as thin and elongated vascular profiles with unidentifiable lumen. Hence, CD34/CD31 double immunofluorescence clearly confirmed the presence of a widespread stromal meshwork of CD34+/CD31− TCs around CD34+/CD31+ blood vessels and CD34−/CD31+ lymphatic vessels (Fig. 3A–C). Since in some organs it has been proposed the existence of a ‘myoid’ subtype of TCs expressing α-SMA^45,46^, human tongue sections were further subjected to CD34/α-SMA double immunostaining. We found that CD34+ TCs from the whole tongue stromal compartment did not coexpress α-SMA (Fig. 3D–F). Of note, this analysis also provided additional details on the tissue localization of TCs, either revealing that the TCs enveloping the secretory salivary gland units formed an outer cell layer encompassing the α-SMA+ myoepithelial cells (Fig. 3D) or confirming that perivascular TCs constituted the tunica adventitia of arterioles immediately outside the α-SMA+ vascular smooth muscle cells (Fig. 3E,F). In addition, lingual TCs were clearly immunonegative for c-kit/CD117 (Fig. 3G–I). Indeed, c-kit/CD117 immunoreactivity was detected solely in oval/round-shaped mast cells that frequently were in close relationship with the cytoplasmic processes of CD34+ TCs (Fig. 3G–I). Keeping into account that the coexpression of CD34 and PDGFRα cell surface antigens is presently considered the most reliable immunophenotypic trait of TCs^1,2,9,10,19–22,44^, we considered crucial to carry out CD34/PDGFRα double immunofluorescence staining. As shown in Fig. 4A–F, the whole CD34+ interstitial cell meshwork of the human tongue was also PDGFRα+, thus further supporting that it consisted of TCs.

**Figure 5:**
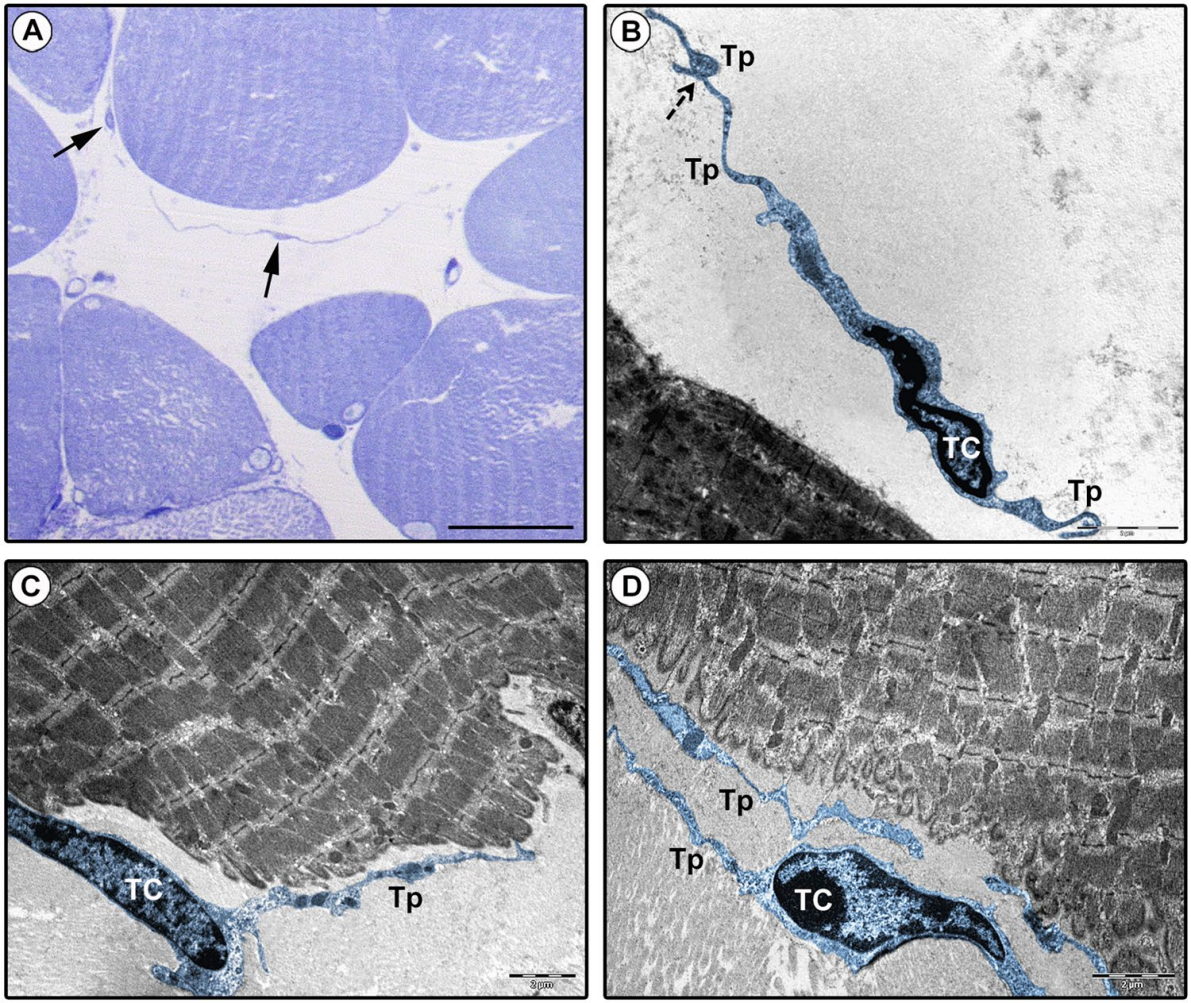
Ultrastructural identification of telocytes (TCs) in human tongue muscle interstitium. (**A**) Semithin sections stained with toluidine blue and observed by light microscopy. Spindle-shaped cells with very long and thin moniliform cytoplasmic processes (arrows) are observed in the stroma surrounding skeletal muscle fibers. (**B–D**) Tongue muscle ultrathin sections stained with UranyLess and bismuth subnitrate solutions and observed by transmission electron microscopy. TCs (digitally colored in blue) are ultrastructurally identifiable as interstitial cells with telopodes (Tp), namely cytoplasmic prolongations with a moniliform silhouette due to the alternation of thin segments (podomers) and expanded portions (podoms). TCs may display a spindle-shaped, oval or piriform cell body mostly occupied by the nucleus. Telopodes are often interconnecting (**B**, dashed arrow) and in close relationship with skeletal muscle fibers (**C,D**). Scale bar: 25 µm (**A**), 2 µm (**B–D**).

Finally, though the limited availability of archival epoxy resin-embedded samples did not allow an electron microscopy investigation of the entire lingual stromal compartment, we could at least confirm the existence of TCs in the tongue muscle interstitium according to relevant ultrastructural identificative criteria^1^ (Fig. 5A–D). Lingual TCs displayed a spindle-shaped, oval or piriform cell body mostly occupied by a large nucleus surrounded by a scarce cytoplasm (Fig. 5B–D). Telopodes appeared as long, slender and convoluted cytoplasmic processes abruptly emerging from the TC body and exhibiting a moniliform morphology conferred by the alternation of podomers and podoms (Fig. 5B–D). In agreement with the results obtained by CD34 immunohistochemistry (Fig. 2D–F), telopodes were often captured in close relationship with skeletal muscle fibers in electron micrographs (Fig. 5C,D).

## Discussion

Although we have recently witnessed the identification and thorough description of TCs/CD34+ stromal cells at different anatomic sites in human beings and other vertebrates^1,2,5–10,16,19,23,40,47,48^, the localization of these peculiar interstitial cells still remains unmapped in many organs. Hence, the present morphologic investigation falls into this scenario and expands our human microscopic anatomy knowledge being the first to demonstrate that TCs also reside in the heterogeneous tissues structuring the tongue, a complex muscular organ found in the vertebrate mouth^49,50^. Indeed, we revealed that spindle-shaped TCs/CD34+ stromal cells displaying distinctive moniliform/varicose prolongations (*i.e*. telopodes) constitute a previously unrecognized cell meshwork spanning in the whole stromal compartment of the normal human tongue, from the basement membrane underneath the stratified squamous epithelium to the underlying lamina propria and the interstitium of the deeper striated muscle.

This study was primarily designed to comprehensively examine TCs by light microscopy through both classic CD34 immunohistochemistry and a number of double immunofluorescence reactions that allowed an in-depth characterization not only of the TC antigenic profiles but also of their specific tissue locations and relationships with neighboring cells. In particular, as exhaustively documented by numerous reports from our and other research groups that have described TCs in different organs^1,2,6,9,10,19–22,44^, we found that also in the human tongue the CD34+/PDGFRα+ immunophenotype can be considered a reliable TC hallmark. Our findings also reinforce the growing notion that CD34/CD31 double immunofluorescence should be carried out in this kind of studies as a useful tool to unequivocally differentiate CD34+/CD31+ endothelial cells of blood vessels from the CD34+/CD31− TCs^6^, that are often concentrated in perivascular locations and may even organize as cell components of the vascular adventitial layer. Moreover, although some authors have pointed out that the thin endothelium of lymphatics might be mistaken with the slender silhouette of TCs^51^, here we clearly show that this is unlikely by using immunohistochemistry since there is no immunophenotypic overlap between CD34−/CD31+ lymphatic endothelial cells and CD34+/CD31− TCs. On the basis of certain similarities in the ultrastructural morphology of TCs and interstitial cells of Cajal (ICC) as testified by the former name of TCs (*i.e*. ICC-like cells)^3^, it has been reported that TCs from some organs may share with the ICC also the expression of c-kit/CD117, though subsequent researches either failed to confirm those findings or demonstrated that TCs are negative for c-kit/CD117 in other organs^1–4,6,9,10,37,38,52^. Similarly, here we show that lingual TCs do not express the c-kit/CD117 marker, whose immunoreactivity was detectable only in oval/round-shaped mast cells frequently observed in the close vicinity of the TC cytoplasmic processes. In addition, by means of CD34/α-SMA double immunostaining we could exclude the presence of a ‘myoid’/‘myofibroblast-like’ TC subtype in the tongue stromal compartment. In fact, all TCs within tongue tissues appeared to be CD34+/α-SMA−, and some TCs were concentrated just on the outside of the CD34−/α-SMA+ myoepithelial cell layer surrounding the secretory acini of lingual salivary glands. Of note, these findings are quite similar to those recently reported in the human testis, where CD34+/α-SMA− TCs were seen to form a peritubular outer layer adjacent to an inner layer of CD34−/α-SMA+ ‘myoid’ cells^9^. On the contrary, the existence of α-SMA+ ‘myoid’ subtypes of TCs has been suggested in the interstitial space of organs of the urinary system, such as the kidney and urinary bladder^45,46^. However, it is worth noting that the aforementioned studies^45,46^ did not employ CD34/α-SMA double immunofluorescence and, therefore, it cannot be excluded that such α-SMA+ spindle-shaped cells referred to as ‘myoid’ TCs might rather correspond to myofibroblasts, which are well known to express α-SMA^53^. Even though we frankly acknowledge that a comprehensive ultrastructural analysis of human tongue stromal space was not possible because of the unavailability of specimens of the lamina propria, it is remarkable that we could confirm the presence of cells ultrastructurally identifiable as TCs within the tongue muscle interstitium in the same locations previously disclosed by light microscopy.

As already discussed, the main goal of the present work was to determine morphologically/immunophenotypically the presence of TCs within the tongue as a hitherto unexplored organ, and therefore we recognize that further analyses will be necessary to definitely shed lights on the precise functions of lingual TCs. Indeed, it should be considered that at present only a few studies have demonstrated with certainty the role of TCs in some organs^2,34,54–56^, and such paucity of functional data may be in part ascribed to the fact that protocols for TC isolation are only at early stages, thus hampering reliable *in vitro* investigations. Nevertheless, we can assume that lingual TCs might play a variety of functions based either on their specific microanatomic locations or on the multiple physiologic roles that have been hypothesized for the TCs in previous works^1,2,4,6,30,40^. As proposed in a number of organs^1,4,6,31,32,40^, it can be speculated that the unique meshwork of TCs extending from the entire lamina propria to the stromal space of the underlying skeletal muscle might have a supportive role during both morphogenesis of the human tongue and post-natal shaping of its extraordinarily complex three-dimensional anatomic structure, that represents a great challenge in the context of regenerative medicine^50,57^. The lamina propria fibrous connective tissue provides support and nutrition to the lingual epithelium and, likely, the TC networks bordering the basal epithelial side and surrounding blood and lymphatic vessels may be important mediators of such functions. Even in the underlying tongue muscle interstitium, TCs were distributed to intimately cover both muscle fibers and vascular and nervous structures, making conceivable their involvement in the regulation of muscular trophism and activity. Though we found that lingual TCs are immunonegative for the c-kit/CD117 antigen, we cannot exclude that a subpopulation of these cells might express other stemness markers, possibly representing relatively immature cells/progenitors with regenerative potential^6,58,59^. Of note, the identification of TCs in the tongue connective tissue and striated muscle, that at variance with the mesenchyme-derived tissues in which these cells have previously been analyzed are known to arise embryologically from cranial neural crests^60,61^, may raise some interesting questions on the TC embryologic origin, which is worth of future investigations. Taken into account that a recent study has identified subepithelial TCs as a crucial source of Wnt molecular signals without which intestinal crypt stem cells cannot properly proliferate and renew^54^, it is tempting to speculate that the TC plexus located just beneath the lingual epithelium might perform similarly. Hence, those TCs could be engaged in intercellular signaling supporting the turnover of epithelial cells covering the tongue surface as well as of specialized cells harbored in the taste buds. In such a context, it is worth mentioning that Wnt signaling seems to exert a fundamental role in supporting tissue integrity during tongue development^62^. Consistent with previous reports on TCs in human exocrine pancreas, parotid gland and labial salivary glands^21,63,64^, we observed that TCs encircle the lingual salivary secretory structures, which might suggest that they are involved in the regulation of glandular functionality in cooperation with the neighboring α-SMA+ myoepithelial cells. Lastly, in line with previous observations in both physiologic and pathologic conditions^1,21,65^, the arrangement of a subset of lingual TCs to precisely delimit and separate subepithelial lymphoid aggregates from the surrounding tissue may be in favor of a participation of those cells in local immunosurveillance.

## Conclusions

In summary, the present findings on the human tongue add to a growing body of data published over the past ten years and strengthen the notion that it is time to reconsider our knowledge of the human microscopic anatomy by recognizing TCs as a distinctive stromal cell identity. However, further studies should be devoted to definitely prove the multiple proposed TC functions and to decipher the mechanisms mediating the cross-talk between TCs and other cell types. Clearly, the ‘strategic’ TC locations that we herein highlighted within the stromal space of the normal human tongue represent the essential groundwork for upcoming functional studies, as well as for future investigations of TCs in different tongue pathologies.

## Methods

### Paraffin-embedded human tongue specimens

Cases were retrieved from the archival files of the Institute of Histopathology and Molecular Diagnosis, Careggi University Hospital, Florence, Italy. A total of 5 consecutive patients (4 females and 1 male, mean age 55 years, range 35–71 years) treated for oral cavity squamous cell carcinoma between March and December 2017 were selected. The main criterion for inclusion was the presence of abundant non-pathologic tissue around the tumor. Clinical and pathologic data were retrospectively collected. All patients were treated with complete surgical excision of the tumor with wide margins. Cervical lymph node dissection was also performed in 4 cases based on clinical and imaging evaluation, and all resulted negative. Normal tissue for the study was selected more than 5 mm far from the infiltrative edge of the tumor. This numerical criterion was applied because tissue that is at least 5 mm or more from invasive carcinoma is considered free of tumor cells when the adequacy of surgical margins is evaluated in the pathologic report^66,67^. One paraffin-embedded normal tongue tissue block per case was selected with informed consent to prepare tissue slides for histochemical and immunohistochemical analyses. The study was carried out in accordance with the Declaration of Helsinki and the approval of the institutional review board of Careggi University Hospital, Florence, Italy.

### Histochemistry

Tongue sections (3 µm thick) from each paraffin-embedded tissue block were deparaffinized and routinely stained with hematoxylin and eosin to confirm the normal tissue appearance (*i.e*. absence of any obvious histopathologic feature).

### Immunoperoxidase immunohistochemistry

CD34 immunohistochemistry on paraffin-embedded human tissue sections (3 µm thick) was carried out following a standardized protocol employing the ready-to-use UltraVision™ Detection System reagents (Anti-Polyvalent HRP, catalog no. TP-125-HL; Lab Vision, Fremont, CA, USA) that has been described in detail in previous reports from our research group on TC investigation in a variety of human organs^9,10,17,21,44,65,68^. Briefly, after deparaffinization, tongue tissue sections were subjected to heat-mediated antigen retrieval in 10 mM sodium citrate buffer (pH 6.0) followed by inactivation of endogenous peroxidases. Tissue slides then underwent blockade of non-specific antibody binding sites and overnight incubation at 4 °C with a primary mouse anti-human CD34 antibody. Detailed information on the antibody source and dilution is provided in Table 1. Negative controls were performed by overnight incubation of serial sections with isotype-matched and concentration-matched irrelevant mouse IgG (Sigma-Aldrich, St. Louis, MO, USA). Antigen-antibody complexes were revealed by sequentially applying to tissue sections biotinylated secondary antibodies, streptavidin peroxidase reagent and 3-amino-9-ethylcarbazole (AEC, catalog no. TA-125-SA; Lab Vision) chromogenic solution. After hematoxylin counterstaining, tissue slides were mounted and observed under a Leica DM4000 B microscope equipped with a Leica DFC310 FX 1.4-megapixel digital color camera and the Leica software application suite LAS V3.8 (Leica Microsystems, Mannheim, Germany).

**Table 1:** List of primary antibodies, their source and working dilutions. Abbreviations: IHC, immunohistochemistry; IF, immunofluorescence.

Primary antibody	Host species	Catalog no.	Producer	Dilution
anti-CD34	Mouse	M7165	Dako, Glostrup, Denmark	1:50 (IHC, IF)
anti-CD31	Rabbit	ab28364	Abcam, Cambridge, UK	1:50 (IF)
anti-α-SMA	Rabbit	ab5694	Abcam, Cambridge, UK	1:100 (IF)
anti-c-kit/CD117	Rabbit	A4502	Dako, Glostrup, Denmark	1:200 (IF)
anti-PDGFRα	Goat	AF-307-NA	R&D Systems, Minneapolis, MN, USA	1:100 (IF)

### Double immunofluorescence

Paraffin-embedded human tissue sections (3 µm thick) were subjected to double immunofluorescence combining mouse with either rabbit or goat primary antibodies as reported elsewhere^9,10,19,21,44,65,68^. Briefly, tongue sections were deparaffinized with xylene, rehydrated, unmasked in sodium citrate buffer (10 mM, pH 6.0), and treated with a glycine solution (2 mg/ml) to quench autofluorescence signals before starting the immunostaining procedure. After blockade of non-specific antibody binding sites by applying a solution of 1% bovine serum albumin in PBS for 1 hour at room temperature, tissue slides were incubated overnight at 4 °C with a mixture of mouse and rabbit or goat primary antibodies diluted in blocking solution. Details on primary antibody sources and dilutions are shown in Table 1. As negative controls, irrelevant isotype-matched and concentration-matched mouse, rabbit and goat IgG (Sigma-Aldrich) were applied in parallel to serial sections. The day after, the immunoreactions were revealed by using the following fluorescent-dye conjugated secondary antibodies diluted 1:200 in blocking solution: Alexa Fluor-488-conjugated donkey anti-mouse IgG for CD34, Rhodamine Red-X-conjugated goat anti-rabbit IgG for CD31, α-SMA and c-kit/CD117, and Alexa Fluor-568-conjugated donkey anti-goat IgG for PDGFRα (all from Invitrogen, San Diego, CA, USA). After nuclear counterstaining with 4′,6-diamidino-2-phenylindole (DAPI), the immunolabeled slides were examined with a Leica DM4000 B microscope (Leica Microsystems). Green, red and blue channel fluorescence images were acquired with a Leica DFC310 FX 1.4-megapixel digital color camera equipped with the LAS V3.8 software (Leica Microsystems). Overlay images were reconstructed using the free-share ImageJ software (NIH, Bethesda, Maryland, USA; online at http://rsbweb.nih.gov/ij).

### Transmission electron microscopy

Semithin and ultrathin sections (2 μm and ~70 nm thick, respectively) from archival epoxy resin-embedded human tongue muscle specimens were processed according to standardized protocols as detailed elsewhere^9,10^. TCs and telopodes detected in electron microscopy images were digitally colored in blue using Adobe Photoshop CS6 software (Adobe Systems, San Jose, CA, USA).

## Data Availability

All relevant data are within the paper.

## References

[1] Cretoiu SM, Popescu LM (2014). Telocytes revisited. Biomol. Concepts.

[2] Cretoiu D, Radu BM, Banciu A, Banciu DD, Cretoiu SM (2017). Telocytes heterogeneity: From cellular morphology to functional evidence. Semin. Cell. Dev. Biol..

[3] Popescu LM, Faussone-Pellegrini MS (2010). TELOCYTES - a case of serendipity: the winding way from Interstitial Cells of Cajal (ICC), via Interstitial Cajal-Like Cells (ICLC) to TELOCYTES. J. Cell. Mol. Med..

[4] Faussone Pellegrini MS, Popescu LM (2011). Telocytes. Biomol. Concepts.

[5] Wang J, Jin M, Ma WH, Zhu Z, Wang X (2016). The history of telocyte discovery and understanding. Adv. Exp. Med. Biol..

[6] Marini M, Rosa I, Ibba-Manneschi L, Manetti M (2018). Telocytes in skeletal, cardiac and smooth muscle interstitium: morphological and functional aspects. Histol. Histopathol..

[7] Cretoiu D, Cretoiu SM, Simionescu AA, Popescu LM (2012). Telocytes, a distinct type of cell among the stromal cells present in the lamina propria of jejunum. Histol. Histopathol..

[8] Popescu LM (2011). Identification of telocytes in skeletal muscle interstitium: implication for muscle regeneration. J. Cell. Mol. Med..

[9] Marini M (2018). Reappraising the microscopic anatomy of human testis: identification of telocyte networks in the peritubular and intertubular stromal space. Sci. Rep..

[10] Rosa I, Marini M, Guasti D, Ibba-Manneschi L, Manetti M (2018). Morphological evidence of telocytes in human synovium. Sci. Rep..

[11] Xiao J, Bei Y (2016). Decoding telocytes. Adv. Exp. Med. Biol..

[12] Cismasiu VB, Radu E, Popescu LM (2011). miR-193 expression differentiates telocytes from other stromal cells. J. Cell. Mol. Med..

[13] Zheng Y (2014). Protein profiling of human lung telocytes and microvascular endothelial cells using iTRAQ quantitative proteomics. J. Cell. Mol. Med..

[14] Zheng Y (2014). Comparative proteomic analysis of human lung telocytes with fibroblasts. J. Cell. Mol. Med..

[15] Sun X (2014). Differences in the expression of chromosome 1 genes between lung telocytes and other cells: mesenchymal stem cells, fibroblasts, alveolar type II cells, airway epithelial cells and lymphocytes. J. Cell. Mol. Med..

[16] Díaz-Flores L (2014). CD34+ stromal cells/fibroblasts/fibrocytes/telocytes as a tissue reserve and a principal source of mesenchymal cells. Location, morphology, function and role in pathology. Histol. Histopathol..

[17] Marini M, Manetti M, Rosa I, Ibba-Manneschi L, Sgambati E (2018). Telocytes in human fetal skeletal muscle interstitium during early myogenesis. Acta Histochem..

[18] Rusu MC (2016). Telocytes of the human adult trigeminal ganglion. Cell. Biol. Toxicol..

[19] Vannucchi MG, Traini C, Manetti M, Ibba-Manneschi L, Faussone-Pellegrini MS (2013). Telocytes express PDGFRα in the human gastrointestinal tract. J. Cell. Mol. Med..

[20] Zhou Q (2015). Cardiac telocytes are double positive for CD34/PDGFR-α. J. Cell. Mol. Med..

[21] Alunno A (2015). Telocytes in minor salivary glands of primary Sjögren’s syndrome: association with the extent of inflammation and ectopic lymphoid neogenesis. J. Cell. Mol. Med..

[22] Banciu A (2018). Beta-Estradiol Regulates Voltage-Gated Calcium Channels and Estrogen Receptors in Telocytes from Human Myometrium. Int. J. Mol. Sci..

[23] Cretoiu SM, Cretoiu D, Popescu LM (2012). Human myometrium - the ultrastructural 3D network of telocytes. J. Cell. Mol. Med..

[24] Gherghiceanu M, Popescu LM (2012). Cardiac telocytes – their junctions and functional implications. Cell Tissue Res..

[25] Cretoiu D, Xu J, Xiao J, Cretoiu SM (2016). Telocytes and their extracellular vesicles—Evidence and Hypotheses. Int. J. Mol. Sci..

[26] Cismaşiu VB, Popescu LM (2015). Telocytes transfer extracellular vesicles loaded with microRNAs to stem cells. J. Cell. Mol. Med..

[27] Marini M, Ibba-Manneschi L, Manetti M (2017). Cardiac telocyte-derived exosomes and their possible implications in cardiovascular pathophysiology. Adv. Exp. Med. Biol..

[28] Fertig ET, Gherghiceanu M, Popescu LM (2014). Extracellular vesicles release by cardiac telocytes: electron microscopy and electron tomography. J. Cell. Mol. Med..

[29] Faussone-Pellegrini MS, Gherghiceanu M (2016). Telocyte’s contacts. Semin. Cell. Dev. Biol..

[30] Edelstein L, Fuxe K, Levin M, Popescu BO, Smythies J (2016). Telocytes in their context with other intercellular communication agents. Semin. Cell. Dev. Biol..

[31] Bani D (2010). Telocytes as supporting cells for myocardial tissue organization in developing and adult heart. J. Cell. Mol. Med..

[32] Sanches BDA (2017). Telocytes play a key role in prostate tissue organisation during the gland morphogenesis. J. Cell. Mol. Med..

[33] Popescu LM (2011). The tandem: telocytes–stem cells. Int. J. Biol. Biomed. Eng..

[34] Albulescu R (2015). The secretome of myocardial telocytes modulates the activity of cardiac stem cells. J. Cell. Mol. Med..

[35] Díaz-Flores L (2015). Human resident CD34+ stromal cells/telocytes have progenitor capacity and are a source of αSMA + cells during repair. Histol. Histopathol..

[36] Ibba-Manneschi L, Rosa I, Manetti M (2016). Telocyte implications in human pathology: an overview. Semin. Cell. Dev. Biol..

[37] Richter M, Kostin S (2015). The failing human heart is characterized by decreased numbers of telocytes as result of apoptosis and altered extracellular matrix composition. J. Cell. Mol. Med..

[38] Manetti M (2013). Evidence for progressive reduction and loss of telocytes in the dermal cellular network of systemic sclerosis. J. Cell. Mol. Med..

[39] Boos AM (2016). The potential role of telocytes in tissue engineering and regenerative medicine. Semin. Cell. Dev. Biol..

[40] Bei Y, Wang F, Yang C, Xiao J (2015). Telocytes in regenerative medicine. J. Cell. Mol. Med..

[41] Zheng L (2018). Transplantation of telocytes attenuates unilateral ureter obstruction-induced renal fibrosis in rats. Cell. Physiol. Biochem..

[42] Ricci R (2018). Telocytes are the physiological counterpart of inflammatory fibroid polyps and PDGFRA-mutant GISTs. J. Cell. Mol. Med..

[43] Nicolescu MI (2016). Telocytes in exocrine glands stroma. Adv. Exp. Med. Biol..

[44] Marini M (2017). Telocytes in normal and keratoconic human cornea: an immunohistochemical and transmission electron microscopy study. J. Cell. Mol. Med..

[45] Rusu MC, Mogoantă L, Pop F, Dobra MA (2018). Molecular phenotypes of the human kidney: Myoid stromal cells/telocytes and myoepithelial cells. Ann. Anat..

[46] Vannucchi MG, Traini C, Guasti D, Del Popolo G, Faussone-Pellegrini MS (2014). Telocytes subtypes in human urinary bladder. J. Cell. Mol. Med..

[47] Aleksandrovych V, Walocha JA, Gil K (2016). Telocytes in female reproductive system (human and animal). J. Cell. Mol. Med..

[48] Ibrahim D, Gaber W, Awad M (2019). Temporospatial localization of telocytes during esophageal morphogenesis in rabbit. Acta Histochem..

[49] Iwasaki S (2002). Evolution of the structure and function of the vertebrate tongue. J. Anat..

[50] Sanders I, Mu L (2013). A three-dimensional atlas of human tongue muscles. Anat. Rec. (Hoboken)..

[51] Rusu MC, Hostiuc S (2019). Critical review: Cardiac telocytes vs cardiac lymphatic endothelial cells. Ann. Anat..

[52] Ceafalan L, Gherghiceanu M, Popescu LM, Simionescu O (2012). Telocytes in human skin–are they involved in skin regeneration?. J. Cell. Mol. Med..

[53] Powell DW (2000). Myofibroblasts: paracrine cells important in health and disease. Trans. Am. Clin. Climatol. Assoc..

[54] Shoshkes-Carmel M (2018). Subepithelial telocytes are an important source of Wnts that supports intestinal crypts. Nature.

[55] Yang J, Li Y, Xue F, Liu W, Zhang S (2017). Exosomes derived from cardiac telocytes exert positive effects on endothelial cells. Am. J. Transl. Res..

[56] Jiang XJ, Cretoiu D, Shen ZJ, Yang XJ (2018). An *in vitro* investigation of telocytes-educated macrophages: morphology, heterocellular junctions, apoptosis and invasion analysis. J. Transl. Med..

[57] Chi JJ, Haughey BH (2014). Tongue transplantation. Curr. Otorhinolaryngol. Rep..

[58] Galiger C (2014). Phenotypical and ultrastructural features of Oct4-positive cells in the adult mouse lung. J. Cell. Mol. Med..

[59] Bojin FM (2011). Telocytes within human skeletal muscle stem cell niche. J. Cell. Mol. Med..

[60] Parada C, Chai Y (2015). Mandible and tongue development. Curr. Top. Dev. Biol..

[61] Parada C, Han D, Chai Y (2012). Molecular and cellular regulatory mechanisms of tongue myogenesis. J. Dent. Res..

[62] Zhu XJ (2017). A Wnt/Notch/Pax7 signaling network supports tissue integrity in tongue development. J. Biol. Chem..

[63] Nicolescu MI, Popescu LM (2012). Telocytes in the interstitium of human exocrine pancreas: ultrastructural evidence. Pancreas.

[64] Nicolescu MI, Bucur A, Dinca O, Rusu MC, Popescu LM (2012). Telocytes in parotid glands. Anat. Rec. (Hoboken)..

[65] Milia AF (2013). Telocytes in Crohn’s disease. J. Cell. Mol. Med..

[66] Alicandri-Ciufelli M (2013). Surgical margins in head and neck squamous cell carcinoma: what is ‘close’?. Eur. Arch. Otorhinolaryngol..

[67] Shah AK (2018). Postoperative pathologic assessment of surgical margins in oral cancer: A contemporary review. J. Oral Maxillofac. Pathol..

[68] Manetti M, Rosa I, Messerini L, Ibba-Manneschi L (2015). Telocytes are reduced during fibrotic remodelling of the colonic wall in ulcerative colitis. J. Cell. Mol. Med..

